# Book Reviews

**Published:** 1817-11

**Authors:** 


### CRITICAL ANALYSIS
### *OF RECENT PUBLICATIONS,*
IN THE
### DIFFERENT BRANCHES OF PHYSIC, SURGERY, AND MEDICAL PHILOSOPHY.

---

*A Picture of the present State of the Royal College of Physicians of London; containing Memoirs, biographical, critical, and literary, of all the resident Members of that learned Body, and of the Heads of the Medical Boards, with some other distinguished Professional Characters: to which is subjoined an Appendix, or Account of the different Medical Institutions of the Metropolis, Scientific and Charitable, with their present Establishments.* 8vo. pp. 548. Sherwood and Co.

THE most interesting part of the present work is the biography of living characters. It will be urged that such can never be impartial. We would ask, Where is the impartiality in the biography of the deceased? But, in a compilation like the present, we have one particular advantage: without any breach of candour, we may presume that the materials are furnished by the characters themselves. This affords, if not a certain, at least the best, source of correctness in some points, and shows, at the same time, how men wish to be described. The filling-up the Picture must, of course, be left to the compiler; but it is not likely to be unsatisfactory to the parties. Under this impression, we shall gladly seize the opportunity of offering the *amende honorable* of two writers, who, it now appears, were jointly engaged in a publication which, at one time, seemed to threaten destruction to every honest endeavour at the improvement of medicine.

"DR. BIRKBECK, *Licentiate of the Royal College of Physicians, and Physician to the Aldersgate Dispensary.*

" Literary talents and strong connexions at all times command success with a professional character, and the respectable individual now before us is proceeding with rapid strides to occupy that place in city practice which a Fothergill and a Lettsom have held before.

" Dr. Birkbeck is a native of Settle, in Yorkshire, where his family resides. His father was a banker, and his brother pursues the same respectable mode of acquiring wealth. They all belong to the sect of Friends, the only sect whose mode of religion is all internal, and forms, what all religion ought to be, the silent passing

 interchange interchange between the individual and the Deity, without form or ostentation, and whose general conduct in society is marked by the meek demeanour of the humble Jesus more than any other. Dr. Birkbeck received the first rudiments of education at Digglesworth school, and, after a sufficient proficiency in general literature, having selected medicine as his professional pursuit, he was sent to Edinburgh. Here he took his degree, and soon after began his career in the metropolis.

"As a young physician cannot, at first, have his time fully employed by real professional occupation, it is fortunate where he possesses a literary turn, as it leads, in the mean time, to real professional improvement, for it directs his attention in literature merely to professional subjects, which is acquiring the experience at a cheap rate. Dr. Birkbeck accordingly availed himself of this, but, possessing that acumen of mind which is more apt to follow its own opinion than go by the sentiments of others, he took an active part in the London Medical Review, and commenced the bold part of a critic on his brethren. His object was particularly directed against those who aimed at what he conceived an improper desire of popularity, a conduct which, though proper in a critic in some cases, tends to stifle the best principles of action in the human mind, if carried on indiscriminately. Indiscriminate censure, however, seemed to be the prevailing object of this work. It breathed nothing of the mild spirit of the friend, or the gentle manner of the sect, and it soon sunk a victim to its own severity, without pleasing the public, or convincing the unfortunate authors, who felt its malignancy, of their supposed errors.

"Soon after his settlement, Dr. Birkbeck was appointed physician to the Aldersgate Dispensary, the duties of which he discharges with much attention and talent. He is a member of several of the medical institutions of the metropolis, and, on the whole, an able and deserving character."

"DR. T. BATEMAN, *Licentiate of the Royal College of Physicians, and Physician to the Carey-street Dispensary.*

"Though the professional character receives a dignity and consequence from literature, yet professional success is not always proportioned to the extent of such acquirements. The present individual we consider as a learned and literary physician.

"Dr. Bateman is a native of Northumberland, and, from his natal soil, at a fit age, was sent to Edinburgh to attain his professional knowledge. Here he continued the usual academical period, and took his degree at this celebrated seminary, with that approbation which a diligent student who looks forward to eminence in life will always deserve. He then repaired to the metropolis, and, after a course of the hospitals, commenced his career in practice. Like every young physician who professes literary acquirements, his first object was to employ these in the laudable improvement of his profession, and he accordingly has connected himself with the leading ing periodical publications of the day. It is the misfortune of one so engaged, to see matters too often through a jaundiced medium: not that we wish to apply this particularly to the present individual."

The remainder of this life is confined to Dr. Bateman's connexion with the Edin. Med. Journal, and also with the late Dr. Willan. The asperity of some reviews in the former is thus easily accounted for. The connexion with Dr. Willan, having been *sub judice*, becomes rather a matter for the lawyers than the faculty.

But, though we are ready to admit these *confessions* as some atonement for the wanton severity of that expired journal—the London Medical Review, and for the more severe critiques in the respectable publication from a sister metropolis, we are far from considering them as *apologies* for either. If there is a department of science in which truth is more important than in all others, it is medicine; because in all others she may ultimately prevail, and maintain justice. The same may happen in medicine; but, in the meanwhile, a mistake in medicine is irretrievable. What may be sport to the wanton or malicious critic, or afford food to the hungry gazetteer, may be death to thousands. Let us, however, dismiss this painful subject, and attend only to the work before us.

Several lives of defunct physicians will be found in our Collectanea. The subjoined Appendix consists of a long Preface concerning the privileges of the College, and their unfair usurpations; an Introduction, containing an apology for offering the lives of living characters; a London Medical Directory, containing a medical and almost statistical Topography of London,—like most others, somewhat too circumstantial for the means in the power of any individual to arrive at,—a concise view of the Royal College of Physicians, the Royal College of Surgeons, the Society of Apothecaries, the Medical Society of London, the Medico-Chirurgical Society of London, the Linnæan Society,—next the charitable establishments, comprehending the Hospitals, Dispensaries, Truss Societies, Vaccine Establishments, and Veterinary College.

Such are the contents of this volume, which, with all its imperfections on its head, we prognosticate will be a source of much amusement to the present generation, and, in future times, frequently become a book of reference, though, perhaps, not of complete authenticity.

*Edinburgh*

### *Edinburgh Medical and Surgical Journal,* No. LII. October, 1817.

Art. I.—*Case of Injury of the Spinal Cord.* By John Gordon, M.D. Fellow of the Royal College of Surgeons, Edinburgh.

This case is well worth recording, as it contains a more minute description of the effect of fractured vertebræ than is usually met with. We think the practice of applying leeches somewhat tame, where the consequences are so soon and so much to be dreaded. We should have preferred cupping-glasses or bold venæsection, or both. There is, indeed, no reason to suppose either would have been useful; but, if any thing was attempted, it should have been with a boldness and decision equal to the danger. *Audacter ferramentum imprimere debet, ut agat aliquid.*

Art. II.—*Cases of Typhus Fever, with Observations on the Nature and Treatment of that Disease.* By J. C. Pritchard, M.D. F.L.S. and F.W.S. Physician to the Infirmary and to St. Peter's Hospital, Bristol.

This paper abounds with just observations; but there is a want of attention to language which surprised us greatly in such a writer. To several very just remarks concerning a deep-rooted prejudice in the public, and even in some of the profession, against blood-letting in continued fever, a note is appended, complaining of the unbecoming language and conduct of two practitioners in a case of epilepsy, for which the author advised blood-letting.

On this occasion we are forced to remark, that, if philosophers, and such we consider Dr. Pritchard, indulge themselves in the use of loose terms, when discussing so important a subject as the treatment of a disease, they must not wonder if men of inferior acquirements seize the only advantage they have over so powerful an antagonist; if they urge that bleeding is improper in typhus, and that epilepsy, being a nervous disease, is likely to be exasperated by evacuants of any kind. Had we been honoured with this communication, we should not have scrupled to return it to the author, with a few hints for his better consideration: as it is, we can only offer them for his government in future, or for our own correction if we are found in an error. To the term *continued* fever we can have no objection; but where was the necessity of introducing the word *typhus.* If this term relates to the infectious property of the disease, every practitioner knows that infectious and contagious fevers from similar sources sources will, in different subjects, require a different treatment. If it relates to the nature of the fever, either that fever was the typhus of authors, or we should have been informed in what it differed: and, if that difference was so great as to require a totally different treatment, it should have had a different name, or some reason should have been given why the name was retained. In the case of epilepsy, too, we ought to have been informed whether the complaint came under the description of chronic or acute; a distinction as old as Aretæus, and the inattention to which among the moderns has brought into disgrace so many remedies for this formidable disease. We have dwelt thus long on a subject the importance of which has so often been urged by us, because we particularly wish that a physician whom, from his former writings, we so highly respect, should be aware, that, whilst he uses the terms Typhus and Epilepsy in so loose a manner, he is one among the number who, with the best intentions, and, in other respects, the soundest judgment, is perpetuating a practice the ill consequences of which he so justly deprecates.

There is another subject on which we enter with much satisfaction, when we reflect on the respectability of the character whom we are addressing.

"After having attentively noticed (says Dr. P.) the symptoms and progress of contagious fever, in a great number of cases which have fallen under my observation, I am very much disposed to believe, that there is no such distemper as what Dr. Armstrong, with some other authors, denominates *Simple* Typhus. I have scarcely ever witnessed a case of this fever, in which there was not some internal organ that appeared to labour more severely than the rest of the system, and exhibited symptoms which I was disposed to attribute to some local congestion, or inflammatory action; and in the great number of instances these symptoms have been nearly unequivocal. I believe the doctrine respecting fever, maintained by Dr. Clutterbuck and some others, gives too confined a view of the nature of this disease. The facts adduced by Dr. Beddoes seem to authorise the inference, that, in a great number of cases, proofs of inflammation are more clearly discoverable in the stomach, lungs, or other viscera of the thorax or abdomen, than in the brain. Yet I cannot but coincide, on the whole, more nearly with Dr. Clutterbuck's opinion than with Dr. Beddoes's, so far, at least, as to regard the general affection of the constitution in typhus as depending on local disease. This opinion I know to be contrary to the most prevalent doctrine in medical schools and among medical authors; yet, as many false notions have long continued to be current from the effect of their previous ascendency, I do not consider this as a proof that my own observations are at variance with the results of general experience. There is one writer, however, who has taken the same side of this question, question, which I am disposed to hold; and it will be seen that the facts I have to adduce tend, as far as their evidence goes, to confirm the opinion which he has maintained respecting the nature and treatment of continued fever. I scarcely need add, that the author to whom I allude is Dr. Mills of Dublin, to whom the profession and the public are much indebted.

"I shall hasten to insert, in as brief and condensed a manner as possible, some notes referring to a few of the cases of typhus, which I have lately treated in St. Peter's Hospital; but I wish, in the first place, to state one or two considerations, which are favourable to the notion that this fever is a symptomatic rather than an idiopathic disease.

"I am persuaded, that, if any physician, who would divest himself of the influence of previous opinion, should attentively examine a number of patients labouring under typhus, he would, in almost every case, be naturally led by the symptoms to infer that there must be some particular organ severely affected, in which was the primary seat of the disease. The idea of some deeply-seated inflammation giving rise to a train of constitutional symptoms, is much more consistent with probability and the general analogy of facts in pathology, than that of a general derangement of the whole constitution occurring independently and primarily. So repugnant, indeed, to probability has the latter opinion appeared on a closer view, that most of those adventurers in medical theory who have framed hypothesis to account for the phenomena of fever, have thought it proper to refer the primary affection to some particular structure,—some of them fancying the nervous, others the vascular, system, to sustain the first shock.

"The opinion which will probably be allowed to be most consistent with the general tenor of pathological observations, will be supported, in a large majority of cases, by an appeal to facts; for it is conceded by all, that the instances are very numerous in which typhus is found, on dissection, to have been connected with local disease. It was long ago observed by Riverius, that acute and malignant fevers *very rarely* happen without the inflammation of some viscus; and this assertion is repeated even by Beddoes, who allows that it has been confirmed 'in every single epidemic where search has been made, and often without search.' Why do we persist in declaring the morbid appearances which manifest themselves, and which seem to be adequate to account for the previous phenomena, to be only accidentally connected with the disease? We have only to extend the same determination to other maladies, and we immediately deprive ourselves of the whole of the resources derived for the improvement of our art from morbid anatomy.

"Such has been the general evidence which anatomical investigation has afforded, and if this evidence has not been uniform, we must take into consideration the very imperfect manner in which these examinations have often been conducted. With respect to those cases which terminate in recovery, and do not give us an opportunity of anatomical investigation, we may observe, that they are chiefly chiefly such as have been treated from the beginning by those means which are calculated to subdue visceral inflammation; and that the more regularly these measures have been pursued in the early stage of the disorder, the greater is the chance of recovery. In my own practice, I have constantly observed, that the most successful measures were those that were founded on the supposed presence of some local inflammation.

"It is true, that cases of typhus fever occur in which no symptoms appear, that may be considered as giving unequivocal proof of the existence of topical affection. But here it is surely more philosophical to reason from the known to the unknown, and, comparing these doubtful cases with others concerning which there is no doubt, to infer, as a *probable* fact, the existence of those morbid causes which, in the majority of cases, we ascertain. Examples are not wanting to show, that deeply seated inflammations of the viscera may exist for a considerable time, and even occasion the death of the patient, without ever giving rise to symptoms that enable the medical attendant to detect the real nature of the malady. A case was lately mentioned to me by Dr. Craufuird, of Clifton, which fell under his observation thirty years ago, the circumstances of which strikingly illustrate this remark. A man was seized with the usual symptoms of typhus, and all the appearances which characterize that disease took place in their regular course, nor was the least suspicion entertained of the existence of any other complaint, or of any thing uncommon in the nature of the case. The patient lived upwards of three weeks, and, after death, when his body was examined, a sac full of pus was found adhering to the coats of the duodenum, near the pylorus."

All this leads to several questions, and to some of which the author does not seem perfectly aware. We need hardly say, that it leads us to consider the word typhus as a cover for our ignorance of the nature or cause of many fevers;—a term, therefore, which a philosopher should never use unless he precisely states his meaning. But it involves a question older than Celsus, and extremely well discussed before his time, namely, that all fever is only the effect of some local disease. That all violent fever is usually attended by some local affection, cannot be questioned; and that the part affected depends much on the constitutional peculiarity of the patient, the season of the year, or the climate. The effects of the two former were extremely well understood by Sydenham, and traced in the different years, and at different seasons, with an accuracy which must immortalize his memory. In our own times, it has been further illustrated by the assistance of morbid anatomy, and by our better acquaintance with tropical fevers, and probably would have been universally attended to, but for the unhappy attempt at a nosological arrangement of fevers in common with other diseases. It is not, however, our intention to admit that all fevers are attended with violent local affection. Even in cases which we have examined *post mortem*, we have frequently found no local affection sufficient to occasion death. As to the effusion on the brain, we have never had an opportunity of examining a subject after fever without it; but in very few instances with suppuration or adhesions sufficient to produce death; or with more effusion than appears to us sufficient to induce the pain in the beginning of fever, and the dulness of the senses towards and during convalescence, until the effused fluid is absorbed.

Dr. P. afterwards remarks, that, even in tropical fevers, local affections are usually discovered. We believe, with much more certainty than in colder regions, especially in the army, consisting chiefly of the natives of the north, in the flower of youth, and in an unnatural state of animation.

After a few other remarks, very judicious, but we can hardly say new, some cases follow, in which some patients were sparingly bled with leeches, others more copiously by the arm, according to their previous condition and mode of life.

"Some of the above cases (continues Dr. P.) were so short in their progress towards recovery under the use of the evacuants administered, that the peculiar symptoms of the typhoid state had scarcely time to develop themselves; and a doubt may hence arise in the minds of some readers, whether they were genuine cases of typhus or not. In order to be convinced that they were such, it was sufficient to witness the circumstances of the origination of the disease in every single instance. I believe there was scarcely one patient whose malady could not be distinctly traced to infection. As a proof of the virulence of the contagion, I may mention, that, at the beginning of the period through which it prevailed, a man who assisted in carrying to the grave the body of a patient who died under this fever, was seized with rigors immediately after his return, and expired on the third day."

By this are we to understand that contagion was sufficient proof of *typhoid* character without *typhoid* symptoms, or what are we to understand by the perpetual recurrence of this unexplained word? If we are to understand contagion, the consideration is important, as directing us in the means of prevention. But, though we thus discover the cause, does this lead in the least to the mode of treatment? Small-pox is contagious; but is every fever from such a cause to be treated alike? On the whole, we are much pleased with the author's practice, excepting that we are quite of opinion, with Dr. Kinglake,* that blisters, in these cases, and espe-

* See page 102 of this volume.

cially, cially, as Sir Gilbert Blane* has remarked, blisters in the immediate vicinity of the inflamed part are often worse than useless. We could wish, also, that Dr. Prichard would look a little further backward than the medical gentlemen he has quoted: highly respectable as they are, it should be recollected that R. Jackson and Sutton were much before them in this mode of treating fevers, equally infectious and more violent than those described by Dr. Prichard.

*(The other Articles in our next.)*

---

*Medico-Chirurgical Transactions, published by the Medical and Chirurgical Society of London.* Vol. VIII. Part I.— Longman and Co. 1817.

These Transactions begin now to assume a kind of official form, the Society having expressed its intention of producing a yearly volume. It is not less certain that it consists of men who have the largest opportunities of practical knowledge, and who are not less respectable for scientific attainments. But it unfortunately happens, in every branch of medicine, that such men have but little leisure, and, for the most part, less inclination, to write. The first difficulty might seem a sufficient apology were it universal; the second may often be imputed to the sage answer of Lucullus's wealthy veteran—"*Vadat qui zonam perdidit.*" It is, however, much to the public advantage that a rival Society to that of Bolt-court has been established. Highly respectable as the last part of the Memoirs of that venerable institution must be universally considered, the papers were principally medical. These "Transactions" are, with very few exceptions, chirurgical, and, though chiefly produced by the junior members, are none of them without their share of merit. The first involves a question concerning early amputation, which has of late come frequently before us.

*Report of the State of the Wounded, on board His Majesty's Ship Leander, in the Action before Algiers; extracted from a Letter from* D. Quarrier, M.D. Surgeon to the Leander, *to the Commissioners for Transports.* Communicated by Sir Gilbert Blane, Bart.

"Herewith I enclose a Report of the wounded on-board this ship, by which you will perceive that the Leander has suffered most severely in this arduous conflict; many of the wounds were inflicted by large round, and double-headed or bar shot; others by grape, langrage, and musquetry, and some few by splinters; but we were

---

* Paper on Tracheitis in Medico-Chirurg. Trans. vol. vi. p. 147

 in in a great measure secured from the latter by our being almost in contact with the shore, and no accident whatever occurred on board, but by the direct fire of the enemy. All our amputations were performed immediately, without waiting for re-action; and it may be necessary to observe, that, though many of the men were carried down with their limbs torn from them; others with the most severe lacerations and fractures; and one young officer in particular, with the spine of the ileum, and all the anterior abdominal muscles torn away, exposing the contents of the abdomen; yet in no instance could we perceive the dreadful perturbation and constitutional shock so frequently described by authors on gun-shot wounds, until some time after the injury had been received; and I have every reason to conceive, that amputation having so promptly followed the wound, was the only effectual means of saving many from its baneful influence, even under the very unfavourable circumstances in which we were placed. Indeed, gentlemen, no language can pourtray the horrors of the Leander's cock-pit for a period of thirteen hours. Sixty-five men were wounded, and several killed, by the first and second broadsides; two poor boys were most dreadfully burnt by a red-hot shot blowing up the cartridge, which one of them was carefully guarding. The small space occupied for their accommodation was instantly crowded to excess: without air; panting for breath; bathed in a most profuse perspiration, and unable to stand upright, these men were to be attended to; water! water! was the incessant cry; most fortunately an abundance had been provided, and the women supplied it liberally. I have already said, gentlemen, that no language can describe the horrors of this scene, but you may figure to yourselves the condition of the miserable sufferers in the black hole at Calcutta, and you will have a correct picture of our condition. Under these disadvantages and difficulties, our operations were performed; and the poor patients were afterwards exposed to the double danger of being trampled on by those who were rushing forward for relief. We could not place them on the lower deck, as many had been wounded there, and the shot were coming in very rapidly. To illustrate what I have observed respecting the non-appearance of that peculiar derangement of the sensorium, which is said always to attend wounds inflicted by large cannon-shot, I shall proceed to give you the following examples.

"Captain Willson, of the Royal Marines, had both his limbs torn away by a double-headed shot; and David Barry, a seaman, had both his thighs torn off by a cannon-ball. Amputation was immediately performed very high up. They lived some hours, and were perfectly sensible until within a few moments of their death.

"Timothy Sullivan, seaman, had his left thigh most cruelly lacerated, the bone having been fractured up to its head, the nerves and blood-vessels torn asunder. The crural artery was readily secured at the groin. His right arm was fractured, and he had a wound in the breast. Still he continued sensible, and made the most earnest supplications that I should operate on him. I declined it until

Francis

Francis Coulthred, who had his right thigh shattered and carried away by a cannon-ball, made such an appeal for him, that he could be no longer resisted. No! said this brave seaman, when he was going to be lifted, I am comparatively easy now; let me entreat you to render some assistance to that poor fellow who is suffering so much, he was a prisoner with me eight years. Amputation was consequently performed at the hip-joint, after the manner of M. Larrey: the vessels were readily secured, and he did not lose four ounces of blood; but, as I had anticipated, he expired almost immediately afterwards. Coulthred, who evinced so much humanity, friendship, and patience, had his thigh amputated, and is now doing well. Six were amputated above the knee, some very high up, where we found the tourniquet useless; but there was no difficulty in restraining the hæmorrhage by the thumb, until the artery was secured. Three were amputated above the elbow, and two in the fore-arm. All bore the operation with great fortitude, and no unfavourable symptom occurred, even although one of them was much injured by a man who was mortally wounded kicking his stump while in the agonies of death. Some of the amputated arms are cured; but I shall send them to the hospital to secure them from their numerous visitors, who, under the idea of kindness, induce them to become irregular, and ruin their health and constitution. One of the boys, so dreadfully scorched, died on the 1st instant; the other is convalescent, but he will be rendered incapable of further service. John Williams died on the 8th: he had been rejected at Portsmouth, where he entered, in consequence of being consumptive, but, having produced an excellent character, and being captain of a gun, the commanding officer requested me to endeavour to cure him: his natural ardour took him on deck in the moment of danger, and his thigh was amputated in consequence of a wound by a cannon-ball. His fine spirits supported him, until nature being exhausted under such an accumulation of disease, he sunk to rise no more."

Seventy of the wounded were comfortably accommodated on the main deck, and in different degrees of convalescence, off Gibraltar, when a "pestilential easterly wind sat in," which induced fever, and every unfavourable aspect in the wounds. A removal produced every good effect in a day's time, shewing that the fever and the unfavourable local symptoms arose, not from any thing generated among the sick, but from atmospherical influence. Several very useful remarks follow, with a reference to Mr. Copland Hutchison's work, lately noticed in our Journal.

The numbers wounded were 122: of these, 76 were discharged fit for duty, 6 invalided, 4 died, and 36 remain on the sick list. The four who died had submitted to the operation a very few hours before death.

*(To be continued.)*

### FOREIGN PUBLICATIONS.

### AMERICA.

*A System of Anatomy, for the use of Students of Medicine.* By CASPAR WISTAR, M.D. Professor of Anatomy in the University of Pennsylvania. 2 vols. 8vo.

[From the New-England Journal of Medicine, &c.]

THE honour of giving to the United States the first American system of anatomy, belongs to the distinguished author of the publication before us. Nor is this its strongest claim to our attention, since it is the production of one, who has long been a celebrated and successful teacher in the first medical school of our country, of one, whose name has long been in the mouth of thousands of physicians, grateful for the fruits of his instructions. With such titles to notice as these, we presume that our tardiness in attending to it, will be attributed to other causes than a want of proper estimation of its importance, or of respect for its scientific and honourable author.

In an attempt to review a work of this nature, it will not be necessary to give an exact account of its contents; and still less so, to enter on a discussion of minute points in anatomy, or doubtful questions in physiology. The proper inquiry is, whether such a publication was called for by the wants of the country; and if so, whether it is well calculated to supply those wants. The first step in such an inquiry seems to be, to take a view, perhaps quite cursory, of the books on anatomy, in common use, especially in the United States.

Our students of medicine have been, we believe, in the habit of employing formerly the system of Cheselden, and of late years, that of the Bells. The precision, simplicity, and comprehensiveness of Cheselden, have continued its popularity longer than is common; and it has passed through two or three editions in this country; but it is too concise for present use, and its physiology is in many parts defective, and in some quite erroneous. The writings of Mr. John Bell, the principal author of the system bearing that name, have caused a great diversity of opinion, as to their merits; for, while none have been more sharply criticised, none have been more generally read. They are evidently the effusions of a man of strong feelings and great talents; who, resolved not to fetter himself with exact forms of expression, indulges the free current of his pen, and does not allow himself to be checked by precision, when it would interrupt the interest, and cool the ardour of his description.

He

He always colours strongly, often with coarseness, and sometimes incorrectly. It may very properly be made a question, whether this kind of style is admissible in anatomy, or any other science composed of descriptions of natural objects. Boyer, so remarkable for his exactness, objects decidedly to the use of figurative or ornamental language, where clearness and precision are so important; and he not only recommends, but gives, through his works, an example of the most severe and unmixed style. Voltaire, he says, complains, and produces it as a proof of bad taste, that eloquence had been introduced even into anatomy. While the justness of these remarks would prevent our recommending Mr. Bell as an exclusive elementary author, we ought to be grateful to him for opening an avenue, which conducts us pleasantly through the rough and forbidding parts of the science, and which invites the advances of many, who would be terrified and repelled by the difficult and barren descriptions of Boyer.

There is a work, which passes under the name of the Edinburgh system of anatomy. This is not destitute of merit; but is defective in some parts, and redundant in others, and altogether a heterogeneous mass, thrown together without method. We have, therefore, no great reason to regret that it has never been reprinted in this country.

Dr. Alexander Monro of Edinburgh, the third professor of anatomy and surgery, bearing that name, has lately made public the course of instruction pursued by his illustrious predecessors and himself. His arrangement is peculiar. He places first the organs of motion, next those of nutrition; namely, the digestive, absorbent, circulatory, and respiratory; then of the voice; afterwards of the urinary and generative functions; next the nervous system, the brain and organs of the senses; and lastly, the distribution of the arteries, veins, nerves, and lymphatics. The description of each apparatus is preceded by a general account of that apparatus, and followed by a collection of relative pathological facts. The latter contains many interesting observations; but cannot be ranked with the collections of Portal; and appears extremely meagre, compared to what we might expect from the storehouse of the Monros. The work, however, appears valuable as well as agreeable, and therefore will be popular; and the author's industry will no doubt be excited to maintain the great name of his family, by improving and enlarging his future editions.

The French anatomists have been distinguished by the production of some excellent treatises. The anatomy of Sabatier differs from the systems of the other French writers,

 in in being a mixture of anatomy and physiology. Notwithstanding this deviation from the custom of the French, it was well received and used as the standard work, until the anatomy of Boyer appeared, which, by its exactness and its rejection of physiology, immediately superseded the former. Boyer's anatomy is a specimen of the most exact and minute description of physical objects. His descriptions of the muscles especially, are extended to a minuteness, which seems unnecessary, and perhaps will not be very acceptable in any country but France. An exact description of the osseous fabric is more useful, because the description of most other parts is continually referring to this; that of Boyer, therefore, if diligently studied, is calculated to form an excellent foundation for the science, and even to create a passion for it. This author is also to be admired for the firmness, with which he maintains his exact method of description against all the obstacles that present themselves. The descriptive anatomy of Bichat, bears strong marks of that powerful genius, that gives an air of novelty to every thing it touches. One would scarcely have thought it possible to add so much interest to so ancient and precise a study, as that of anatomy, by some changes in arrangement, some alterations of nomenclature, and some new ideas, or rather new views of things already known. It is unfortunate that a part only of the descriptive system was the work of Bichat; for he died when about half of it only was accomplished. The remainder was performed by his pupils Buisson and Roux, in a manner, not dishonourable to the reputation of these ingenious men, but which has not the spirit of their master. Bichat did not attempt great innovations in the system of descriptive anatomy, probably because he did not consider them necessary, or did not believe they would be received. He proposed, indeed, a different arrangement, because the old one was extremely defective; but he corrected the nomenclature with a temperate hand, and yet availed himself with so much address of the labours of Chaussier, that, while the nomenclature of the latter is hardly adverted to, but as a curiosity, the changes of Bichat are actually adopted in France, and will be so in England, as soon as his anatomy has received an English dress.*

Professor Soemmerring's book, "De Corporis Humani Fabrica," is valuable as a collection of facts, but intolerable for its style. The Latin translator employed by Soemmerring has involved his ideas in very long and obscure sen-

* This anatomy is now translating into English.

tences.

tences. The construction of a sentence of six or eight lines is as much as we can commonly embrace with convenience, in the vernacular language; but this translation presents us sentences of half a page, and even of a complete octavo page, of difficult Latin. This might be easily remedied in an English translation, to which the work has every claim, as well as the publications on the eye, ear, the organs of smelling, tasting, &c. all of them possessed of so much merit, that the industry and ingenuity of posterity will with difficulty surpass them.

A novel and beautiful treatise on anatomy may yet be composed, in which the plan would be strictly analytical. Such a system would not, indeed, be calculated for the beginner, because it would require a preliminary knowledge of the elements of the human fabric, and especially of the osseous system. It should commence with a description of the external form and proportions of the body, their differences in individuals and in the sexes. Next would follow an exact examination of the prominences and depressions, which present themselves, particularly about the articulations, and a comparison of them with some of the phenomena, which are the consequences of disease or accident. After this might be described the appearance of the skin in various healthy individuals, its alterations in colour and texture from disease; and then its internal structure. To this would succeed the cellular membrane, the fascia; and, those being supposed to be turned aside, the muscles, arteries, veins, nerves, lymphatic glands, and their connexions and relations to each other, displayed in such lights, as would exhibit them most distinctly and usefully, and, above all, their relations to those parts of the bony fabric, which are most remarkable on the surface of the body. The same plan might be pursued in regard to the organs of the great cavities. The advantages of such a system of anatomy must be obvious. It is the anatomy for practice. It is the anatomy that every one meets when he dissects the dead body. It is the anatomy that every one must picture to his imagination, when he takes up the knife to operate. Many materials for such a system are already prepared by the admirable labours of Cooper, the Bells, Monro, Burns, and Watt.

The work, which is the object of these remarks, was designed by its author, principally for the use of those who attend his course of lectures. Its plan is adapted accordingly, and does not seem to be copied from that of any other writer. The defective and embarrassing division of the subject of anatomy into seven parts, which has been received by almost all anatomical writers, is here quite abandoned. The book is divided into eleven parts. The first contains the osseous system; the second the muscular; third, the ligamentary and synovial; fourth, the brain, spinal marrow, eye and ear; fifth, the integuments, cellular membrane, and skin. The arrangement thus far agrees with that proposed by Bichat; but in the remainder of the work, the author, governed no doubt by convenience for dissection and demonstration, has pursued a different method. In part sixth, he describes the nose, mouth, and throat; in part seventh, the thorax and its contents; in the eighth, the organs of the abdomen and pelvis; ninth, the blood-vessels; tenth, the nerves; eleventh, the absorbent vessels; lastly, an appendix on the blood, and structure of glands, and a glossary of anatomical terms. This plan is perfectly adapted to the circumstances which occur in a course of lectures on anatomy; and, as the design of the author was to make his work an auxiliary to his course, his judgment could not be better exhibited than in such a plan. The lecturer on anatomy is for the most part excluded from the advantages of an exact method, although perhaps there is no science in which they would be more desirable; for a distinct and connected plan of arrangement would enable the student to form some anticipated notion of the parts of the human fabric, and thus dissipate that perplexing obscurity, which arises from the dependance of the knowledge of one part on that of another. To one, also, who has become in some measure acquainted with the whole human system, it is a great pleasure and advantage to look back, through the medium of a good arrangement, and in a single view, to connect the knowledge of all those parts, which it has cost much time and pains to study separately. In point of connexion, the excellence of the arrangement, proposed by Bichat, is very conspicuous. For its foundation it takes the uses or functions of organs; and its principal divisions are laid out conformably to the finest views of agreement and difference in the beings, which compose the organized creation.

In the first and second parts, which treat of the osseous and muscular systems, Dr. Wistar has not considered it necessary to vary his language materially from that of other writers; because he found theirs well accommodated to his purpose. In speaking of the articulations, he, with great justice, questions the utility of the common arrangement of those parts. We would agree with him in rejecting their nomenclature and arrangement altogether. The terms of *symphysis* and *suture*, it might be necessary to retain, because they have been applied to particular parts, namely, the connexion of the bones of the pubes with each other, and that of the bones of the cranium; yet they ought to be considered as denoting, not species, but individual articulations.

As

As for the *synchondrosis*, *synneurosis*, *syssarcosis*, and *synarthrosis*, *gomphosis* and *scindelesis*, and *diarthrosis*, *enarthrosis*, *arthrodia*, *ginglymus*, and *amphiarthrosis*, they are horrible terms, meaning nothing useful; they are a stumbling block to the young student, and a laughing stock to the proficient; they are rarely understood by any one, and no sooner understood than forgotten; in short, they are scarcely explained by two anatomists in the same way, and seem to be of no other use than to enable stupid teachers to astonish ignorant young men.

The third part, which treats on the ligaments, &c. contains many useful additions to our English anatomy. The fourth is peculiarly excellent. The description of the organ of vision is one of the most exact pieces of anatomy we possess; that of the organ of hearing renders this difficult part remarkably clear and intelligible, and at the same time exhibits powers of strong conception and distinct description. The account of the nostrils, mouth, and fauces, is very satisfactory and useful. The organs of the thorax and abdomen are extremely well described. The structure of the lungs, barely made intelligible by other writers, is here explained as if it was intended to be understood; of course it is explained, just so far as our senses are capable of observing it, and nothing is allowed to imagination. This, in truth, is a peculiar excellence in the author of this anatomy, that he never allows himself to ramble from what is positively known, and we therefore always feel secure under his guidance. His description of the digestive apparatus is not less worthy of commendation for its exactness and distinctness.

The style of this work is concise and aphoristical. A style not intended to excite or maintain the reader's interest; but perfectly adapted for more important objects, strength of description, and comprehension of many facts in a small space. Had the author allowed himself to write freely and fluently, he would have swelled the book beyond those limits which are most convenient for the wants of students in this country. If there be an error on either side, it appears to be that of too much conciseness, when we consider that this is, or will be the standard system of the United States.

We give thanks to the author of this work for the industry which he has exerted and the talents he has displayed in its production. It exhibits a perfect knowledge of the science it teaches, a correct taste, a judgment which always discriminates between what is known and what is doubtful; between what is useful and what is superfluous; and all these merits are enhanced by the modesty and want of pretension, which are conspicuous throughout.

### GERMANY.

*Oordalkundige Beschryving van Eenige der Voornaamste Hulkündige Operatien, &c.—Critical Description of some of the principal Surgical Operations performed in the Academical Hospital of Groningen, from November* 1810, *till November* 1815. By P. J. Hendriksz, &c.—Communicated by Dr. Von Embden, of Hamburgh.

The above little work commences with critical illustrations of the operation of lithotomy, and the various methods in which that operation has been performed, from the time of Marianus Sanctus down to Langenbeck. With the latter and Camper, the author recommends an extensive wound in the bladder; in other respects, he makes use of Langenbeck's apparatus with the greatest advantage. The first observations describe the successful issue of the operation performed on a boy of six years of age, who had suffered for four years before the disease was ascertained, and afterwards confirmed by the examination with the catheter. The operation was performed on the 5th of May, after Langenbeck's method, and succeeded so well, that the little patient, who had been previously much emaciated, was restored soon after to his parents, and in the best health. The same method was equally successful in the case of a man pretty far advanced in years.

The second section treats on the operation for the cataract. This operation has ever shared a similar fate with that before-mentioned; each operator had his own peculiar method of operating, instruments either invented or improved by himself, and considered his own as the only safe method and apparatus. The author, for the most part steering clear of each, considers only critically the two principal methods which have been practised, viz. extraction and depression. Both have been practised with equal success, and both had their respective champions among the most respectable of the profession. Dr. Hendriksz, from his own experience, prefers the extraction of the lens or its capsula, to depression. If the anterior, posterior, or whole surface of the capsula is opake, or if it adheres to the iris, at the opening of the pupil, (*synizesis,*) the indication will always be in favour of extraction. Besides, in depression, the patient is always exposed to the danger of its rising again, partly or wholly, and resuming its former situation. The author recommends the instrument invented by Guerin and improved by himself, as particularly useful. The fleam invented by Guerin, and Scarpa's needle for depression, and, in case the lens lens does not come out of its own accord, Daviel's spoon and a pair of pincers to remove the capsula are all the instruments he makes use of. The reason why he prefers the fleam, is the fixed and immoveable condition it gives the eye, in consequence of the excavated ring on its inner peripheric side, without, however, in the least pressing it; it also keeps the eyelids at a distance from each other, retaining the aqueous humour, and, consequently, keeping the cornea tense, and, above all, the secure performance of the incision, by determining its depth, size, and semicircular shape. Of seven patients on whom the author operated in this manner very lately, six recovered entirely. Towards the conclusion of this chapter, he makes some remarks on the proper nature of the true black cataract, which he does not wish to be confounded with that generally termed gutta serena, or amaurosis. "Experience (says he,) has taught us, that the lens may assume a dark, opake, and even black appearance,—a complaint which better deserves the name of amaurosis than a palsy of the optic nerve."

The third section treats of amputations, which, besides a very remarkable observation upon an amputation below the knee-joint, in a general and complete degeneration of the substance of the bone, contains some critical and practical ideas on the extirpation of the joints, and the uncertainty of separating the morbid substance of the bone from the healthy part, &c.

The fourth and last section contains some practical and diagnostical ideas on the character and treatment of sarcocele and testicles enlarged and degenerated by inflammation. Disorders which particularly require the extirpation of the testicles are, according to our author's opinion, either a scirrhous degeneration of their substance, or a suppuration. That scirrhous degeneration, known by the name of sarcocele, is to be carefully distinguished from all other testicular enlargements, and particularly from that swelling and induration which is the consequence of previous inflammation, as the latter affection never, but the former always, requires extirpation. Antecedent inflammation, a gradual hardness, swelling of the affected part to a certain degree, are the symptoms which the author ascribes to an indurated testicle of this description. The nature of sarcocele, on the contrary, he describes to consist in a degeneration of the organic principle of the testicle, specific gravity and hardness, a rough surface, pricking pains, a fixed pain in the spermatic cord, followed by violent pain when the tumour is touched, degeneration of the spermatic cord, varicose distensions of the vessels of the scrotum followed by erysipelatous pelatous inflammation of the latter, itching pain and excoriation of the same, local inflammation of the skin, followed by exulceration, fungus, suppuration, and ichorous secretion, combined with wasting of the whole body, are described as the characteristic symptoms of scirrhus, or, which is the same with our author, of sarcocele.

[In our next we shall give an account of Dr. Mansert's Treatise on Puncturing the Cornea.]

---

*Chemical Amusement, comprising a Series of curious and instructive Experiments in Chemistry, which are easily performed, and unattended by danger.* By Fredrick Accum, Operative Chemist, &c. Crown 8vo. pp. 191; 1817. Callow.

We know not a more useful companion, in the present state of pharmacy, than this little work. Most young men have leisure in the morning, and in order to acquire a practical knowledge of chemistry, and a readiness at operating, they can no longer trust to preparations, which at present many of them receive from Apothecaries' Hall, or some chemist on whom they can depend. Thus, a youth, at the end of his apprenticeship, may have a theoretic knowledge of the principles of chemistry without the least facility at manipulation. These "Amusements," as they are very properly called, will furnish a rational mode of passing part of the morning, and produce a readiness at every experiment, which will be found necessary on many trying and unlooked-for occasions. The operator will require scarcely any instrument which the shop does not furnish, excepting the blow-pipe, which we have recommended on many occasions. We would advise him, for common occasions, to cultivate the habit of using the common blow-pipe with the mouth; but, for artificial gases, nothing can exceed the modern invention.

We shall transcribe only a few experiments, to shew the plan of the whole.

"*Phosphoric Fire Bottle.*—Take a common brimstone match, introduce its point into a bottle containing oxide of phosphorus, so as to cause a minute quantity of it to adhere to it: if the match be then rubbed on a common bottle cork, it instantly takes fire. Care should be taken not to use the same match a second time immediately, or while still hot, as it would infallibly set fire to the oxide of phosphorus in the bottle.

"*Rationale.* The friction on the cork raises the temperature of the oxide of phosphorus, which then inflames and sets fire to the sulphur with which the match is tipped.

"The

"The phosphoric fire bottles may be prepared in the following manner: Take a small phial, of very thin glass, heat it gradually in a ladleful of sand, and introduce into it a few grains of phosphorus; let the phial be then left undisturbed for a few minutes; and proceed in this manner until the phial is full. Another method of preparing this phosphoric bottle, consists in heating two parts of phosphorus, and one of lime, placed in layers, in a loosely-stopped phial, for about half an hour. Another very simple method is the following: Put a little phosphorus into a small phial; heat the phial in a ladleful of sand; and, when the phosphorus is melted, turn it round, so that the phosphorus may adhere to the sides of the phial; and then cork the phial closely."

The following is the elegant experiment of Count Rumford, from which so many economic improvements have resulted:—

"*To render visible the opposite currents into which fluids are thrown, whilst they change their temperature.*—Fill a common eight-ounce phial, or cylindrical glass jar, about two inches or more in diameter, and five or six inches long, with cold water, and diffuse through it a small portion of pulverized amber: let the phial of water be immersed into a tumbler, or larger vessel, containing hot water: this being done, two currents, going in different directions, will be observed in the inner vessel, the one ascending, and the other descending; that is to say, the minute particles of amber, which were diffused through the fluid, and were at rest before the heat was applied to the water in the inner vessel, will be seen in motion: those particles that are situated towards the sides of the glass, or which are nearest to the source of heat, will move upwards, whilst those that are in the centre move downwards; and thus two distinct currents are formed in opposite directions, the central one being directed downwards, and the exterior one upwards. These currents gradually diminish in velocity; and, when the water in the inner vessel has acquired the same temperature as that in the outer one, the particles of amber will again be brought to a state of rest.

"If the position of the two vessels be reversed, namely, if the glass containing hot water be immersed into a vessel containing cold water, the motion of the currents will also be reversed: the particles next to the sides of the glass are thrown into currents directed downwards, whilst the particles in the centre form a current directed upwards. The equilibrium of these two currents will also be restored, when the equalization of temperature of the water within, and that without, has been effected.

"*Rationale.* In the first instance, the caloric of the hot water in the outer vessel is transmitted, by the medium of the glass, to the particles of the water next to the glass in the inner vessel; these particles expand, and are rendered specifically lighter, ascend, and form the outer current. During this process, they gradually part with their caloric to the lateral particles; they become condensed, and thus produce the central current. In reversing the experiment, when the hot phial is surrounded with cold water, the external particles, instead of being heated, lose caloric, or become cooled, and consequently diminished in bulk: their specific gravity is lessened, and therefore they descend; and the central particles, being warmer and specifically lighter, becoming forced up, the currents are reversed.

"To render the experiment more decisive, the lower part of the water may be coloured by tincture of cabbage, or red ink, leaving the upper part uncoloured. If heat be then applied to the bottom part of the glass, the coloured part of the water gradually ascends, and uniformly tinges the whole fluid. Now this certainly can only take place by the actual mechanical interchange of the particles of the water itself. The heat which is applied to the lower strata of the water, becomes specifically lighter than the other particles; it is, therefore, pressed upwards by the adjoining particles, and, being at liberty to move, it changes its place, and is urged up to the surface, viz. in consequence of the fluidity of the body, and the expansion of the separated particles. New particles approach the source of heat, combine with it in their turn, are again displaced, and thus the currents are produced. The rapidity of the motion of the currents (the cause of which is a change in the specific gravity of the fluid, produced by a change of temperature) is in proportion to the change of the specific gravity of the fluid, by any given temperature."

*Rotary Motion of Camphor upon Water.*—Fill a saucer or broad bason with water, and let fall upon it camphor, reduced to the form of coarse sand. The floating particles will instantly begin to move and acquire a progressive rotary motion, which continues for some minutes, and then gradually subsides.

If the water be touched by any substance which is in the slightest degree greasy, all the floating particles briskly dart back, and are, as if by a stroke of magic, instantly deprived of their motion and vivacity.

*Rationale.*—A variety of opinions have been formed concerning this phenomenon. Lichtenberg assigned it to the emanation of an etherial gas from the morsels of the camphor. There was, however, always a certain mysterious caprice in these motions, according to which they sometimes could not be produced; and on other occasions, the motions were instantly stopped when the water was touched with certain bodies, without its being easy to guess the reason. And all these circumstances tended to envelope the phenomenon with obscurity. Venturi was the first who explained the experiment. He was led to the explanation in the following manner:

' Pieces of camphor were cut into the form of small columns, one inch in length; a base of lead was fixed to each column; they were then placed upright in very clean saucers, and pure water poured in, to half the height of the column. Two or three hours afterwards, an horizontal notch was manifest in the column of camphor at the surface surface of the water; and in the course of twenty-four hours, by the notch becoming gradually deeper, the column of camphor was cut in two at the middle. The two pieces of the column, nevertheless, that is to say the lower, which was immersed in the water, and the upper in the air, suffered scarcely any perceptible diminution.

From this experiment, and others made with different pieces of camphor, kept separately in the air, in the water, and at the surface of the water, Venturi deduces, that the most active virtue for dissolving camphor, resides at that part where both the air and the water touch it at the same time.

The camphor at the surface of the water, does nothing, therefore, but dissolve; and, when dissolved at the ordinary temperature of the atmosphere, it is not at first in the state of vapour, as has been thought; it is simply a liquid, which extends itself over the surface of the water; and by this means, coming into contact with a great surface of air, it is afterwards absorbed and evaporated.

The rotary motion of the pieces at the surface of the water, is therefore supposed to be simply the mechanical effect of the re-action which the oily or camphoric liquor, extending itself upon the water, exercises against the camphor itself. If the retro-active centre of percussion of all the jets do not coincide with the centre of gravity, a combined motion of rotation and progression must follow. And, as the departure of the camphoric solution takes place only at the surface of the water, the rotation cannot be effected but round an axis perpendicular to the horizon; and since, in similar bodies of different magnitudes the algebraic ratio of the sides to the mass increases in the inverse duplicate ratio of the sides themselves, the small particles must have proportionally more jets, and must revolve more speedily than the larger. No better explanation has yet been given.'

The experiments are 103 in number, at the end of which Mr. Accum has added "A descriptive Catalogue of his apparatus and instruments employed in experimental and operative chemistry, in analytical mineralogy, and in the pursuits of the recent discoveries of voltaic electricity."

---

